# Ulnar Nerve Palsy in Both-Bone Forearm Fracture in a Pediatric Patient: A Case Report

**DOI:** 10.7759/cureus.28239

**Published:** 2022-08-21

**Authors:** Saeed S Alghamdi, Fahd A Alhejili, Ahmad H Alharbi, Emad A Alzahrani, Salem J Bajuifer

**Affiliations:** 1 Orthopaedic Surgery, Alnoor Specialist Hospital, Makkah, SAU; 2 Orthopedic Surgery, Alnoor Specialist Hospital, Makkah, SAU; 3 Department of Medicine and Surgery, College of Medicine, Umm Al-Qura University, Al-Abdia Main Campus, Makkah, SAU

**Keywords:** elastic nailing, closed fracture, pediatric fractures, ulnar nerve palsy, both bone forearm fracture

## Abstract

Closed fractures of both the radius and the ulna are common in the pediatric age group; however, ulnar nerve palsy is a rare complication with this type of fracture. We present a case of a fracture in both forearm bones in an eight-year-old boy. The patient was admitted for closed reduction and internal fixation. Before surgery, he developed signs of ulnar nerve palsy. The surgery took place under general anesthesia without complications. Postoperative recovery took place with signs of ulnar nerve palsy, and he was discharged the following day. After three months, the ulnar palsy completely resolved, and the fracture had healed. This case shows that a physical examination and ulnar nerve function should be assessed pre- and post-manual manipulation so that the patient can be managed properly. When nerve involvement is noted after manual manipulation of the limb, we recommend surgical intervention and fixation. Controlled studies would allow the development of an algorithm for managing similar cases.

## Introduction

Closed fractures of both the radius and the ulna, also referred to as "both-bone fractures," are common in the pediatric age group [1]. Ulnar nerve palsy, a rare complication [2], can present as deteriorating neurological symptoms. It can be detected at various stages, sometimes immediately (with the injury), post-manipulation, or even at a later date. In cases of worsening neuropraxia [3], MRI can be used to determine the status of the nerve in a case of worsening neuropraxia [3]. Although both-bone forearm fractures are typically managed conservatively [4], Stavrakakis et al. [3] concluded that surgical exploration of the nerve should be performed when palsy is accredited to manual manipulation or when neuropraxia worsens. Herein, we present a case of ulnar nerve palsy after a both-bone fracture in an eight-year-old boy.

## Case presentation

An eight-year-old boy without medical or surgical history presented to the emergency room, referred by his local primary healthcare center after an accidental fall in a park. The patient complained of pain and left forearm deformity but not loss of consciousness or vomiting, and a splint was applied at the primary healthcare facility. The patient denied previous traumas, and his family history was insignificant.

**Figure 1:**
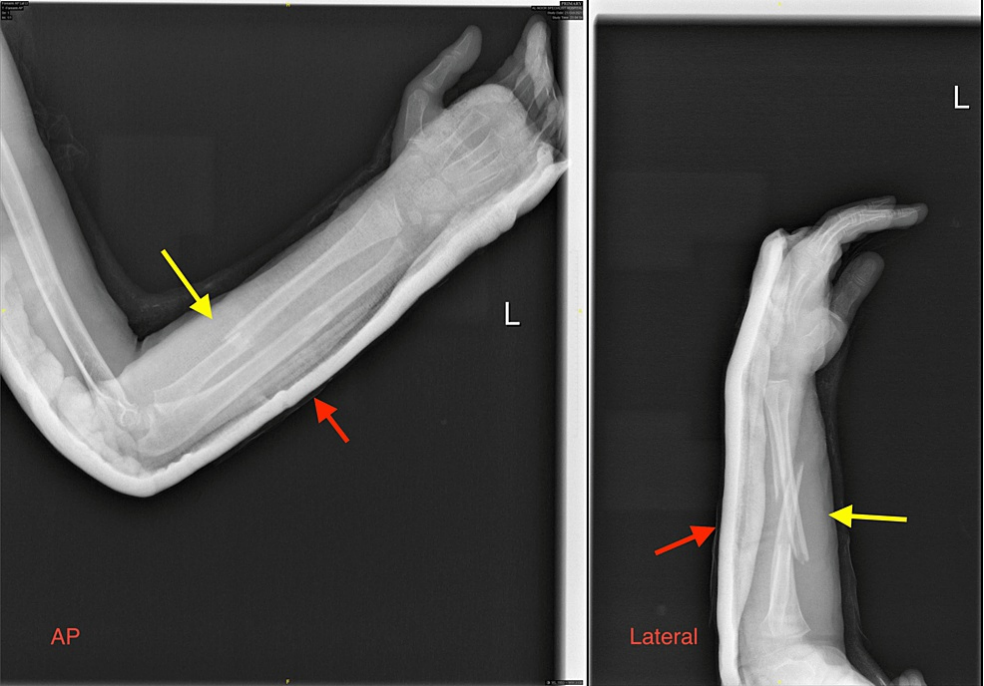
Anterior-posterior and lateral radiographs of the left forearm closed both-bone fracture at presentation. Yellow arrows indicate the fracture site at the left forearm; red arrows indicate the plaster above-elbow back slab.

**Figure 2:**
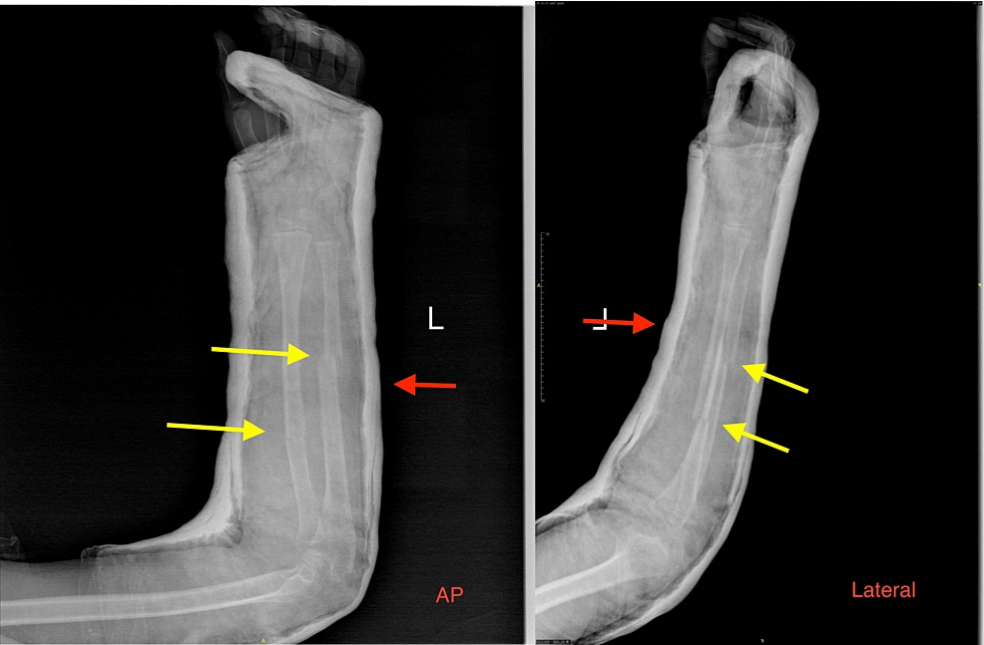
Anterior-posterior and lateral radiographs of the left forearm closed both-bone fracture and plaster above-elbow full cast placed after initial reduction. Yellow arrows indicate the fracture site; red arrows indicate the above-elbow full cast plaster.

Physical examination confirmed a conscious, oriented, well-nourished, and vitally stable patient and that an above-elbow back slab was applied (Figure 1). The neurovascular exam was normal. After removal of the back slab, closed reduction was performed under conscious sedation, followed by placement of a full cast above the elbow. Post-reduction radiographs revealed good alignment with minor displacement (Figure 2).

**Figure 3:**
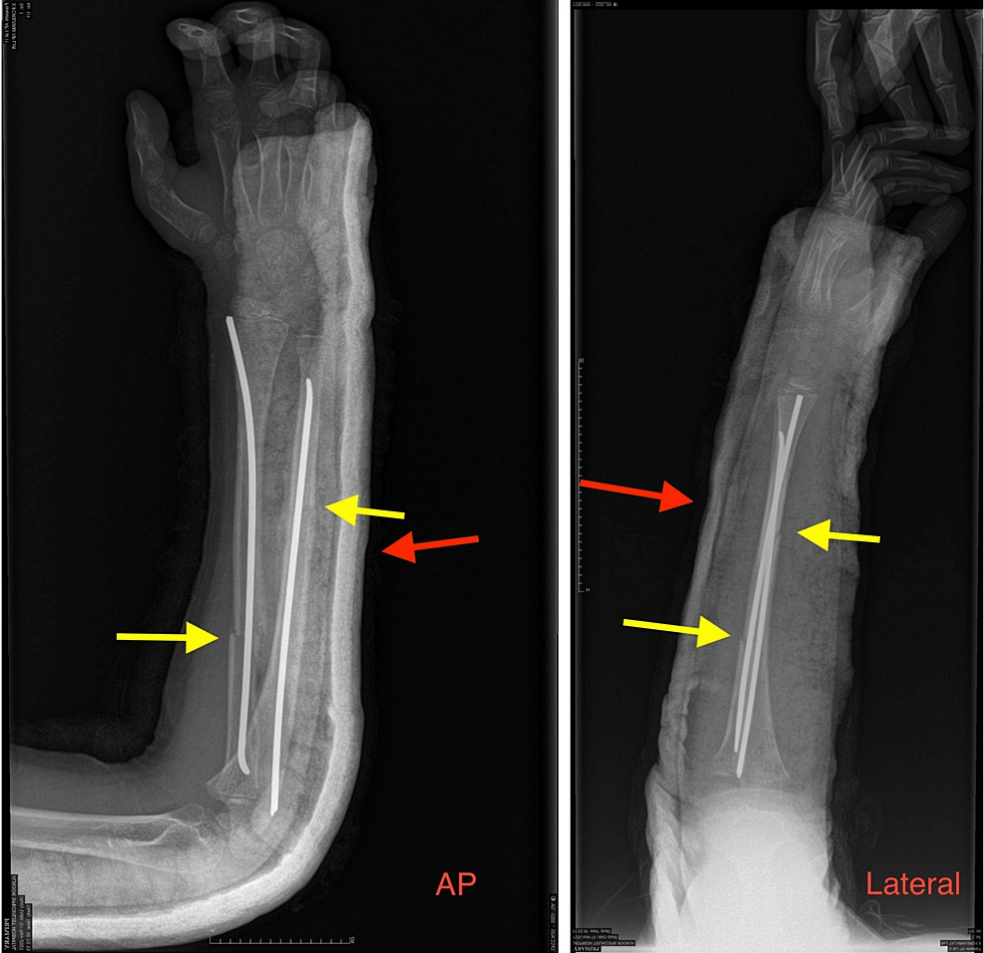
Postoperative anterior-posterior and lateral radiographs of the left forearm showing acceptable reduction and good alignment of both the ulna and radius. Yellow arrows indicate the fracture site at the left forearm; red arrows indicate the above-elbow back slab plaster placed postoperatively.

After consulting with the patient’s family, a closed reduction with elastic nail fixation was determined to be the best treatment. Initial laboratory tests, which included a complete blood count, basic chemistries, and coagulation profile, showed these parameters to be within normal limits. The patient was admitted for surgery. Prior to surgery, however, an examination revealed ulnar nerve palsy indicated by numbness, pain, and tingling in the left hand, with decreased grip strength in the fourth and fifth fingers. Under general anesthesia, closed reduction was performed without complications using a C-arm X-ray machine to assist with internal fixation by elastic nails, placing one retrograde nail, size 3.0 mm, through the distal lateral for the radius, and one antegrade nail, size 3.0 mm, for the ulna (Figure 3).

**Figure 4:**
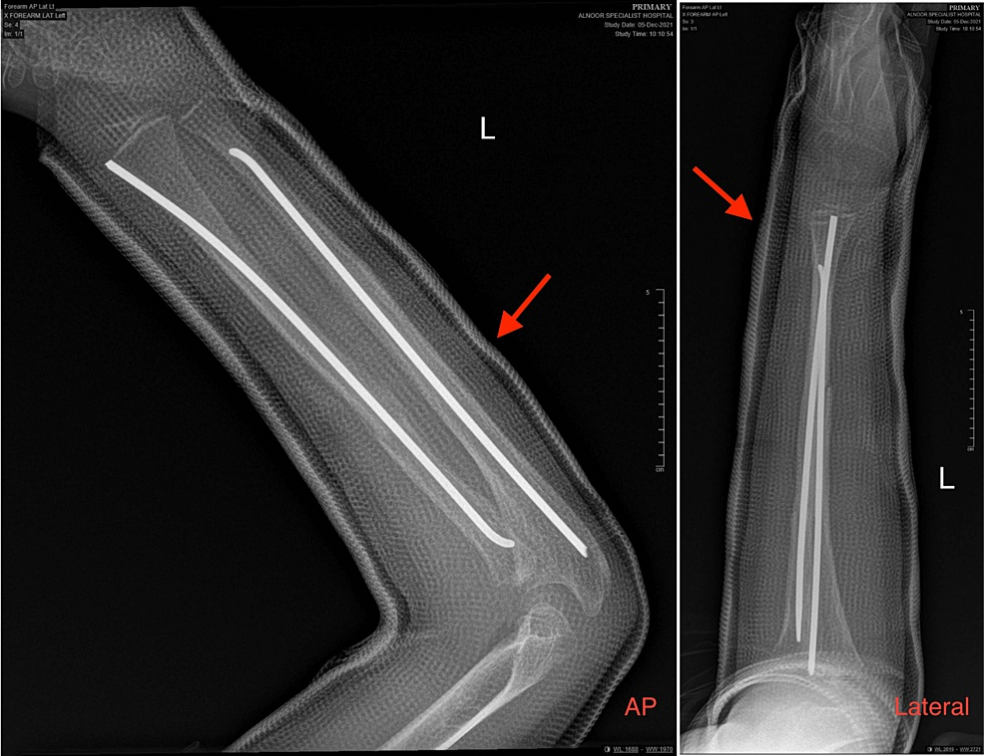
An anterior-posterior and lateral X-ray at six weeks clinic follow-up visit, showing signs of good healing at the fracture site. The red arrow pointing at a full cast plaster placed above elbow level at a previous visit.

**Figure 5:**
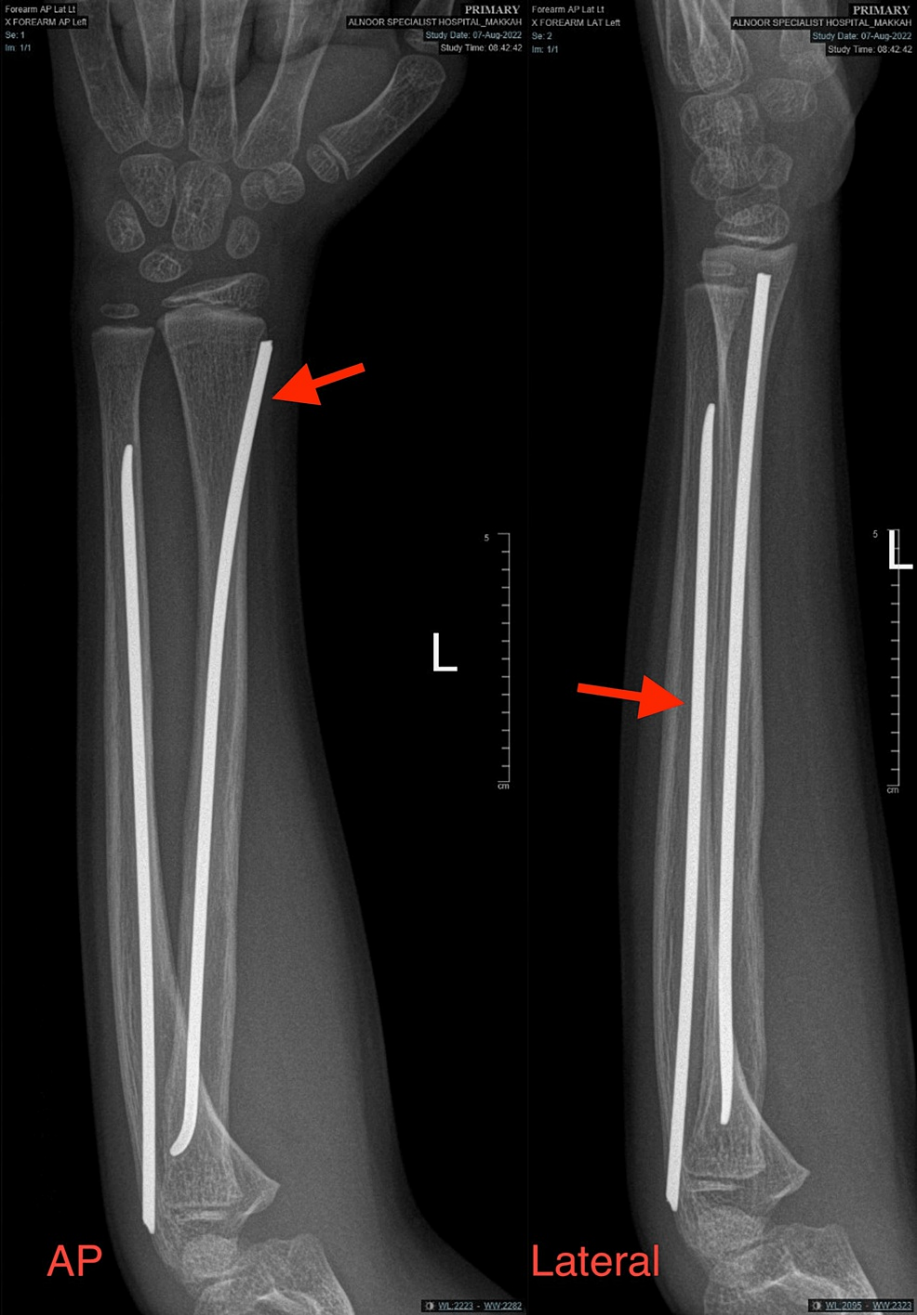
Anterior-posterior and lateral radiographs at the three months follow-up. Full cast plaster removed, complete healing of both bones, the red arrow pointing at the elastic nails used.

Although there were signs of ulnar nerve palsy, the patient was discharged the following day after all instructions were explained to the patient and his family. A follow-up with the pediatric orthopedic trauma clinic was arranged for two weeks later. We followed up at two weeks, six weeks (Figure 4), and three months, finding progressive resolution of signs of ulnar nerve palsy noted by the recovery of numbness and tingling first and followed by grip strength. Three months after surgery, the patient fully recovered from the ulnar palsy and the fracture (Figure 5). The patient was given a follow-up appointment for a checkup and removal of the elastic nails.

## Discussion

Pediatric forearm fractures account for 45% of all childhood fractures [1]. Associated nerve injuries, however, are rare. According to Toor et al. [2] and to the best of our knowledge, the literature provides only 17 cases similar to this one. Both-bone fractures are typically managed conservatively, performing closed reduction followed by casting except when conditions warrant surgical intervention. This includes an unacceptable or lost reduction of the limb, open fractures, fractures in patients with less than two years left to complete growth, neurovascular compromise, and certain pathological fractures [4]. Ulnar nerve palsy can happen as a result of several causes: direct nerve contusion, scar tissue or bone entrapment, or partial nerve laceration [3].

Federer et al. [5] recently published a case series and noted the role of nerve conduction studies in age-appropriate children as an evaluation of nerve function, a baseline for future follow-ups, and as a factor when reconsidering surgery in conservatively managed patients. A multitude of other studies also recommended the use of MRI to best visualize the nerve [3,5,6].

Researchers recommend conservative treatment if a contusion is the cause of nerve pathology; this can be seen prior to manipulation. However, when ulnar nerve injury is the result of manual reduction, surgical reduction is recommended because nerve entrapment or laceration is most likely, as with our patient [2,3]. Moreover, complete anatomical reduction is preferred when surgery is performed so that nerve entrapment or nerve tethering can be avoided [3]. Surgery is also recommended when healing is delayed after conservative management, although no specific time frame is mentioned [7]. Federer et al. [5] concluded that frequent follow-up evaluations (at four to six weeks) are a must, and in cases where physical examination is inconclusive of nerve function, nerve conduction studies and MRI can be used.

## Conclusions

Both-bone forearm fractures are common in the pediatric population, however, associated ulnar nerve injuries are rare; this is why we recommend that physical examination and ulnar nerve function should be assessed pre- and post-manual manipulation so that the patient can be managed properly. When nerve involvement is noted after manual limb manipulation, surgical intervention and fixation are recommended. Regular follow-ups and assessments are recommended no matter the management strategy, and examinations should be performed at each visit to ensure good progression of the symptoms. Further controlled studies would contribute to the development of an algorithm that could be used when managing similar cases.

## References

[1] Rodríguez‐Merchán EC (2005). Pediatric fractures of the forearm. Clin Orthop Relat Res.

[2] Toor R, Antao N, Ghag N (2021). Rare presentation of ulnar nerve palsy in closed both bone forearm fracture in pediatric population. J Orthop Case Rep.

[3] Stavrakakis IM, Daskalakis II, Magarakis GE, Christoforakis Z, Katsafarou MS (2019). Ulnar nerve injuries post closed forearm fractures in paediatric population: a review of the literature. Clin Med Insights Pediatr.

[4] Pace JL (2016). Pediatric and adolescent forearm fractures: current controversies and treatment recommendations. J Am Acad Orthop Surg.

[5] Federer AE, Murphy JS, Calandruccio JH, Devito DP, Kozin SH, Slappey GS, Lourie GM (2018). Ulnar nerve injury in pediatric midshaft forearm fractures: a case series. J Orthop Trauma.

[6] Sneag DB, Curlin J, Saltzman EB, Carlson MG, Lee SK (2021). Role of high-resolution peripheral nerve magnetic resonance imaging in diagnosing median nerve tethering in a case of both-bone forearm fracture in a child. Pediatr Radiol.

[7] Hamdan MQ, Haddad BI, Hawa A, Abdelhamid SS (2019). Ulnar nerve palsy as a complication of closed both-bone forearm fracture in a pediatric patient: a case report. Int Med Case Rep J.

